# Attenuation of *Yersinia pestis fyuA* Mutants Caused by Iron Uptake Inhibition and Decreased Survivability in Macrophages

**DOI:** 10.3389/fcimb.2022.874773

**Published:** 2022-05-04

**Authors:** Yulu Chen, Kai Song, Xin Chen, Ye Li, Ruichen Lv, Qingwen Zhang, Yujun Cui, Yujing Bi, Yanping Han, Yafang Tan, Zongmin Du, Ruifu Yang, Zhizhen Qi, Yajun Song

**Affiliations:** ^1^ State Key Laboratory of Pathogen and Biosecurity, Beijing Institute of Microbiology and Epidemiology, Academy of Military Medical Sciences (AMMS), Beijing, China; ^2^ Lab for Bacteriology, State Key Laboratory of Pathogen and Biosecurity, Beijing Institute of Microbiology and Epidemiology, Academy of Military Medical Sciences (AMMS), Beijing, China; ^3^ School of Basic Medicine, Anhui Medical University, Hefei, China; ^4^ Huadong Research Institute for Medicine and Biotechniques, Nanjing, China; ^5^ Qinghai Institute for Endemic Disease Prevention and Control, Xining, China; ^6^ National Health Commission - Qinghai Co-construction Key Laboratory for Plague Control, Xining, China

**Keywords:** *Yersinia pestis*, iron uptake system, Ybt system, *fyuA*, virulence, pathogenicity

## Abstract

*Yersinia pestis* is the etiological agent of plague, a deadly infectious disease that has caused millions of deaths throughout history. Obtaining iron from the host is very important for bacterial pathogenicity*. Y. pestis* possesses many iron uptake systems. Yersiniabactin (Ybt) plays a major role in iron uptake *in vivo* and *in vitro*, and in virulence toward mice as well. FyuA, a β-barrel TonB-dependent outer membrane protein, serves as the receptor for Ybt. In this study, we examined the role of the *fyuA* gene in *Y. pestis* virulence using different challenging ways and explored the underlying mechanisms. The BALB/c mouse infection assay showed that the virulence of the mutant strains (Δ*fyuA* and Δ*fyuA*_GCAdel_) was lower when compared with that of the wild-type (WT) strain 201. Furthermore, the attenuation of virulence of the mutant strains *via* subcutaneous and intraperitoneal challenges was far greater than that *via* intravenous injection. Iron supplementation restored lethality during subcutaneous challenge with the two mutants. Thus, we speculated that the attenuated virulence of the mutant strains toward the mice may be caused by dysfunctional iron uptake. Moreover, Δ*fyuA* and Δ*fyuA*_GCAdel_ strains exhibited lower survival rates in murine RAW264.7 macrophages, which might be another reason for the attenuation. We further explored the transcriptomic differences between the WT and mutant strains at different temperatures and found that the expressions of genes related to Ybt synthesis and its regulation were significantly downregulated in the mutant strains. This finding indicates that *fyuA* might exert a regulatory effect on Ybt. Additionally, the expressions of the components of the type III secretion system were unexpectedly upregulated in the mutants, which is inconsistent with the conventional view that the upregulation of the virulence genes enhances the virulence of the pathogens.

## Introduction

Iron is indispensable to almost all living things, but its availability is very low (Noinaj et al., 2010). In mammals, iron mainly exists in the form of Fe^3+^-protein complexes (ferritin, transferrin, and lactoferrin), and the concentration of free iron ions does not exceed 10^−18^ M, which is too low to sustain bacterial growth and reproduction (Bullen et al., 1978). Different iron acquisition systems have been identified in various bacterial pathogens (Schaible and Kaufmann, 2004; Braun, 2005; Fetherston et al., 2010; Butt and Thomas, 2017; Dichtl et al., 2019).

*Yersinia pestis*, the causative agent of plague, possesses multiple iron transport systems, including two heme transporters named Hmu and Has, many ATP-binding cassette (ABC) transporters (Yfu, Yfe, and Yiu), several ferrous iron transporters (Yfe, Feo, Fet, etc.), and the yersiniabactin (Ybt) system, which play a major role in the virulence toward mice (Fetherston et al., 2010; Perry et al., 2015). Ybt has been identified in pathogenic *Yersinia* species (*Y. pestis*, *Y. pseudotuberculosis*, and *Y. enterocolitica*), and it serves as a siderophore, a class of low-molecular-weight (<1000 Da) non-protein compounds with high specificity and high affinity for Fe^3+^ (Heesemann, 1987; Heesemann et al., 1993; Perry et al., 1999). Ybt consists of one salicylate, one thiazoline, and two thiazolidine rings, which have six coordinate binding sites for Fe^3+^ (the formation constant for Fe^3+^ is 4 × 10^36^) (Chambers et al., 1996; Gehring et al., 1998; Perry et al., 1999; Miller et al., 2006; Drechsel et al., 1995).

FyuA is a TonB-dependent β-barrel outer membrane receptor that acts as the dual receptor for Ybt and pesticin (Heesemann et al., 1993; Rakin et al., 1994; Lukacik et al., 2012). Ybt can capture Fe^3+^ from lactoferrin and transferrin. Subsequently, the Ybt-Fe^3+^ complex is transported into the cells *via* the outer membrane receptor FyuA. Upon reaching the periplasm, the complex is transported into the cytoplasm by two inner membrane YbtP/YbtQ (Perry and Fetherston, 2011). The TonB system is composed of ExbB-ExbD anchored to the inner membrane and the peripheral lipoprotein TonB. The system provides energy for TonB-dependent outer membrane receptors to transport nutrients, such as iron complexes and vitamin B12 (Schauer et al., 2008). In *Y. pestis*, Ton-box is located at the N-terminus of the FyuA plug domain, which is important for interactions with TonB-ExbB-ExbD (Rakin et al., 1994; Fetherston et al., 1995; Lukacik et al., 2012). *Y. pestis* possessing mutations in either *fyuA* or *tonB* displays similar defects in iron-deficient growth (Perry et al., 2003). Given its importance in transporting ferric iron, FyuA also plays a key role in the virulence of some pathogenic *E. coli* strains and *Yersinia*.

In this study, we constructed Δ*fyuA* (lacking *fyuA*) and Δ*fyuA*_GCAdel_ (GCA three-base deletion at positions 915–917 of *fyuA*) mutants and compared them with the wild-type (WT) strain. Our results revealed that both mutants were less virulent toward HeLa cells and animals, which might be attributed to iron uptake deficiencies in the mutants.

## Materials and Methods

### Bacterial Strains and Culture Conditions

The bacterial strains and plasmids used in this study are listed in **Supplementary Table 1**. Strain 201 belongs to the biovar Microtus and was isolated from *Microtus brandti* in Inner Mongolia, China. This strain is extremely lethal to mice but avirulent to humans (Fan Z, 1995). The strains were grown in Luria-Bertani (LB) broth (pH 7.4) or on LB agar plates at 26°C (*Y. pestis*) or 37°C (*E. coli*). The glycerol-preserved strains were inoculated into 5 ml of LB medium and allowed to be fully activated. The recovered strains were subjected to two consecutive passages, and the second-passage cultures were used in follow-up experiments. Chloramphenicol (Cm) was added to the media at 34 μg/ml when needed.

### DNA Preparation

Plasmids and genomic DNA were extracted using the QIAprep Spin Miniprep Kit (Qiagen, Germany) and QIAamp DNA Mini Kit (Qiagen, Germany) separately in accordance with the manufacturer’s instructions. Polymerase chain reaction (PCR) was performed with an automated thermal cycler (Bio-Rad T100™) using 1×Taq Master Mix (purple) (Biomed, China). The initial denaturation step (94°C, 5 min) was followed by 30 cycles of denaturation (95°C, 30 s), annealing (the temperature was set according to the primers used, 30 s), and extension (72°C, the time was set according to the length of the target gene), with a final extension step (72°C, 5 min).

### Construction and Identification of the Mutant Strains and Complementation Strains

The upstream and downstream homology arms of *fyuA* were amplified from strain 201 using the primer pairs Pre-*fyuA*-F/R and Post-*fyuA*-F/R separately, and the amplification products were purified with QIAquick PCR Purification Kit (Qiagen, Germany). The pDS132 plasmids were digested with *Sph* I-HF (New England BioLabs, USA). The homology arms and digested pDS132 plasmids were ligated using 2×Seamless Cloning Mix (Biomed, China) at 50°C for 15 min, followed by introduction into S17λ*pir* using a Bio-Rad Gene Pulser Xcell Electroporation System (2.5 kV, 25 μF, 200 Ω), thereby generating S17-pDS132-*fyuA* and S17-pDS132-*fyuA*_GCAdel_.

S17-pDS132-*fyuA* and strain 201 were grown in LB medium at 37°C and 26°C, respectively, to an OD_620 nm_ of 0.6–0.8. S17-pDS132-*fyuA* (1.5 *ml*) and 201 (100 μl) cultures were centrifuged at 4500 rpm for 5 min, resuspended in 50 μl of LB medium, and sufficiently mixed, following which the cells were pipetted onto a filter paper (45 μm) attached to the LB plate. The plate was incubated overnight at 26°C. The cells were then eluted with LB, coated on a Yersinia Selective Agar Base (Difco™, USA) plate containing 6.8 μg/ml chloromycetin, and incubated at 26°C for 4–5 days. Subsequently, 5–6 single colonies were selected and placed in LB medium and subjected to a shaking culture overnight at 26°C, coated on LB plates containing 7% sucrose for screening, and then incubated at 26°C for 3 days. PCR using the *fyuA*-seqF/R primers was performed for verification; the amplification products were then confirmed using DNA sequencing.

To construct the complemented strains, a PCR-amplified DNA fragment containing the *fyuA* coding sequences together with 300 bp and 283 bp of its respective upstream and downstream sequences was cloned into pACYC184 that had been digested with *Hin*d III-HF and *Bam*H I-HF (New England BioLabs, USA). The purified PCR products and digested pACYC184 were ligated using 2×Seamless Cloning Mix (Biomed, China) at 50°C for 15 min and then transferred into DH5α cells (Biomed, China). After DNA sequence verification, the recombinant plasmids expressing *fyuA* were introduced into Δ*fyuA* and Δ*fyuA*_GCAdel_ separately, thus generating the complemented mutant strains Δ*fyuA*-Comp and Δ*fyuA*_GCAdel_-Comp. The primer sequences used are shown in **Supplementary Table 2**.

### Growth Curve Determination of *Y. pestis In Vitro*

The *Y. pestis* strains were grown in LB medium at 37°C to an approximate OD_620 nm_ of 1.0 (*ca*. 2 × 10^8^ CFU/ml). The bacterial cultures were diluted 1:20 in a 50 ml Erlenmeyer flask containing small glass beads and 20 ml of fresh LB and then incubated at 37°C with shaking at 220 rpm. The bacterial growth was monitored by measuring the absorbance at OD_620 nm_ at 2 h intervals. The experiment included three independent biological replicates, and the results were expressed as mean ± standard deviation from three independent experiments. The growth curve determination of *Y. pestis* in TMH (0.1 mM FeSO_4_ and 10 μM FeCl_3_) medium (Zahorchak and Brubaker, 1982; Straley, 1986; Drechsel et al., 1995) was performed at 26°C using the same method as above.

### Real-Time Cell Analysis (RTCA) Assay

The HeLa cells were cultivated in Dulbecco’s modified Eagle’s medium (DMEM, Gibco, Thermo Fisher Scientific, USA) containing 10% fetal bovine serum (FBS) at 37°C in a 5% CO_2_ incubator. For this assay, 150 μl of DMEM (Gibco, Thermo Fisher Scientific, USA) containing 10% FBS was pipetted into each well of the E-plate connected to the RTCA iCELLigence system (ACEA Biosciences, San Diego, CA, USA), which was placed at 37°C in a 5% CO_2_ incubator, to measure the baseline. The concentration of the HeLa cells was adjusted to 5 × 10^5^ cells/ml, and 300 μl was pipetted into each well of the E-plate. The E-plate containing the cells was incubated for 30 min at 37°C in a 5% CO_2_ incubator, followed by transfer to the RTCA iCELLigence system, which was maintained under the same condition to obtain a stable baseline (10 h). The harvested *Y. pestis* cells were resuspended in sterile phosphate-buffered saline (PBS) and used to infect the HeLa cells at a multiplicity of infection (MOI) of 10. The cells were incubated, and the cell index (CI) was measured at 2 min intervals.

### Protein Structure and Function Prediction

Iterative Threading ASSEmbly Refinement (I-TASSER, as “Zhang-Server”) (Yang and Zhang, 2015) is a hierarchical approach for protein structure prediction and structure-based functional annotation. Identification of the structural templates from the Protein Data Bank (PDB) (https://www.rcsb.org/) using the multiple threading approach LOMETS, with full-length omics models constructed using iterative template-based fragment assembly simulations is the first step. Insights into the function of the target are then derived by rethreading the 3D models *via* a protein function database, BioLiP (Yang et al., 2013). I-TASSER has been ranked as the No. 1 server for protein structure prediction in recent community-wide CASP 7–14 experiments.

### Mouse Infection

*Y. pestis* was cultivated according to the above method to *ca*. 2 × 10^8^ CFU/ml. The cultures were centrifuged at 4500 rpm for 5 min, washed twice, and diluted in sterile PBS to the desired cell density. The concentration of the bacterial cells was determined *via* dropping on Hottinger’s agar plates. The female BABL/c mice (6–8 weeks old) were provided by Beijing Vital River Laboratory Animal Technology Co. Ltd. [laboratory animal permit no. SCXK (Jing) 2016-0006]. The animals infected with the WT and mutant strains received the injection of 100 μl cultures diluted with PBS to the desired concentration *via* three routes (subcutaneously, intravenously, or intraperitoneally). The mice that needed iron supplementation were injected (subcutaneously or intraperitoneally) with 50 μg of FeCl_2_ diluted in sterile deionized and distilled water. Mortality was recorded continuously for 14 d. GraphPad Prism 5.0 software was used to draw the survival curves. The data were analyzed using log-rank (Mantel–Cox) test, with P < 0.05 considered statistically significant. In this study, all mice were handled as per the Guidelines for the Welfare and Ethics of Laboratory Animals of China.

### Ability of *Y. pestis* to Survive in the Blood and Serum of Mice

Mouse blood and serum were provided by Beijing BioRab Technology Co. Ltd. A total of 950 μl of blood was pipetted into each well of a 24-well plate. The second-passage *Y. pestis* cultures were grown to *ca*. 2 × 10^8^ CFU/ml, centrifuged at 4500 rpm for 5 min, and resuspended in PBS. Subsequently, 50 μl of the bacterial suspension diluted 625 times (*ca*. 3.2 × 10^5^ CFU/ml) was pipetted into a 24-well plate to which 950 μl of blood was added in advance. The 24-well plate was placed at 37°C in a 5% CO_2_ incubator. At 0, 2, 4, 6, and 8 h, the blood was mixed fully by pipetting, and 50 μl of it was pipetted into a 96-well plate to which 200 μl of PBS was added to each well in advance. Thus, a five-fold gradient continuous dilution was created until the number of bacterial colonies could be counted. The number of bacteria in blood was counted by dropping 40 μl of diluted blood onto Hottinger’s agar plates in triplicate. This experiment had three independent biological replicates, and the result was expressed as mean ± standard deviation (n = 3).

### Ability of *Y. pestis* to Survive in RAW264.7 Cells

RAW264.7 cells were maintained in DMEM (Gibco, Thermo Fisher Scientific, USA) containing 10% FBS at 37°C in a 5% CO_2_ incubator. Later, 500 μl of the cells were pipetted into 24-well plates at a concentration of 4 × 10^5^ cells/ml on the day before the infection. The second-passage *Y. pestis* cultures were grown to a bacterial concentration of approximately 2 × 10^8^ CFU/ml and were collected separately by centrifugation and resuspended in sterile PBS. The RAW264.7 cells were then infected with strain 201 at an MOI of 5, and 50 µg/ml of gentamycin was added to the medium to kill the extracellular bacteria after 30 min of infection. At 0.5, 1, 2, 4 and 8 h post infection (hpi), the culture medium from each well was discarded and the cells were thoroughly washed three times in PBS. The infected RAW264.7 cells were then lysed by the addition of sterile 0.1% Triton X-100 for 15 min at room temperature to release the intracellular bacteria. The living engulfed bacteria were counted by dropping the diluted cell lysate onto Hottinger’s agar plates in triplicate. This experiment comprised three independent biological replicates, and the results were expressed as mean ± standard deviation (n = 3).

### RNA Isolation, Sequencing, and Data Analysis

*Y. pestis* WT strain 201 and the Δ*fyuA*_GCAdel_ mutant were grown at 26°C or 37°C in TMH to a bacterial concentration of approximately 2 × 10^8^ CFU/ml, and each sample included three biological replicates. Total RNA was extracted using the PureLink™ RNA Mini Kit (Invitrogen, Thermo Fisher Scientific, USA) and then used for creating a cDNA library and deep sequencing. According to the values of fragments per kilobase of transcript per million mapped reads, the ratio of transcript levels between WT and Δ*fyuA*_GCAdel_ groups was used as the logarithm to the base 2 (twofold change). For example, a twofold change value of 1.0 indicates twofold greater expression of a certain gene. The differential values of at least twofold were applied to analyze the differential expression of genes according to the *Y. pestis* 91001 genome annotation.

### Quantitative Reverse Transcription PCR (qRT-PCR) Analysis

qRT-PCR was used to confirm the results of RNA-seq. RNA samples that were subjected to sequencing were reverse-transcribed to cDNA as templates using SuperSript™ Reverse Transcription Kit (Invitrogen, Thermo Fisher Scientific, USA). The qRT-PCR analysis was performed based on SYBR Green I (Roche) fluorescence using Roche Light Cycler 480. Correlations between the sequencing data and the qRT-PCR results were calculated using linear regression. All primer pairs were designed to produce amplicons with expected sizes of 50–200 bp using the Primer Premier 5.0 software. The primer sequences used are shown in **Supplementary Table 3**.

### Ethics Statement

The animal study was conducted in accordance with institutional guidelines and ethical regulations of the Beijing Institute of Microbiology and Epidemiology.

### Statistical Analysis

All data of the three experimental groups were obtained from three independent experiments and expressed as mean ± standard deviation. If the data conformed to normality and homogeneity of variance, the one-way analysis of variance was performed, and the Student–Newman–Keuls-q test was applied for multiple comparisons. If the data did not conform to normality, the Kruskal–Wallis test was performed, and the comparisons were analyzed using the Nemenyi test. If the data conformed to normality but not to homogeneity of variance, the Brown–Forsythe test was performed. The log-rank (Mantel–Cox) test was performed for survival curve analysis. Statistical significance was set as follows: *, p < 0.05, **, p < 0.01 and ***, p < 0.001.

## Results

### Identification of the Δ*fyuA* and Δ*fyuA*_GCAdel_ Mutants and Determination of Their Growth Curves

**Figure 1:**
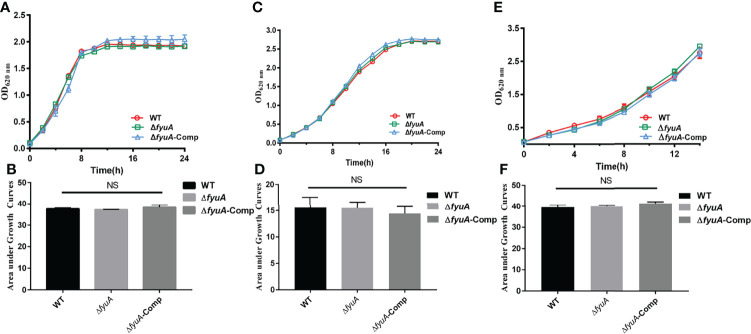
Growth curves of *Y. pestis*. Growth curve of WT, Δ*fyuA*, and Δ*fyuA*-Comp at 37°C in LB **(A)**, at 26°C in defined TMH with 0.1 mM FeSO_4_ **(C)**, or at 26°C in deferrated TMH with 10 μM FeCl_3_ **(E)**. The bacterial growth was monitored by measuring the absorbance at OD_620 nm_ at 2 h intervals. The areas under the growth curve were applied for the statistical analysis of different conditions **(B, D, F)**.

To explore the role of *fyuA* in the virulence of *Y. pestis* biovar Microtus strain 201, the suicide vector pDS132 was used to construct the mutants Δ*fyuA* and Δ*fyuA*_GCAdel_. The corresponding complemented strains were constructed (**Supplementary Table 1**) as described in Materials and Methods. The mutants were identified using PCR and sequencing (**Supplementary Figure 1**). *In vitro* growth curves constructed for strain 201 and Δ*fyuA* in LB medium and TMH (a defined medium) containing different concentrations and types of iron ions showed no significant differences (**Figures 1A–D**). This finding indicated that the loss of *fyuA* had limited effect on the growth of the mutant strains under conditions of abundant iron and nutrition. Thus, phenotypic analysis of the mutants in comparison with the WT strain should not have been influenced by the differences in growth rates. Interestingly, the mutants showed growth rates similar to those of strain 201 in iron-depleted medium (**Figures 1E, F**), which differed from the result of KIM6+ *fyuA* mutant that exhibited retarded growth in iron-deficient medium (Fetherston et al., 2010).

### Attenuation of the Cytotoxicity of the Mutants toward HeLa Cells

**Figure 2:**
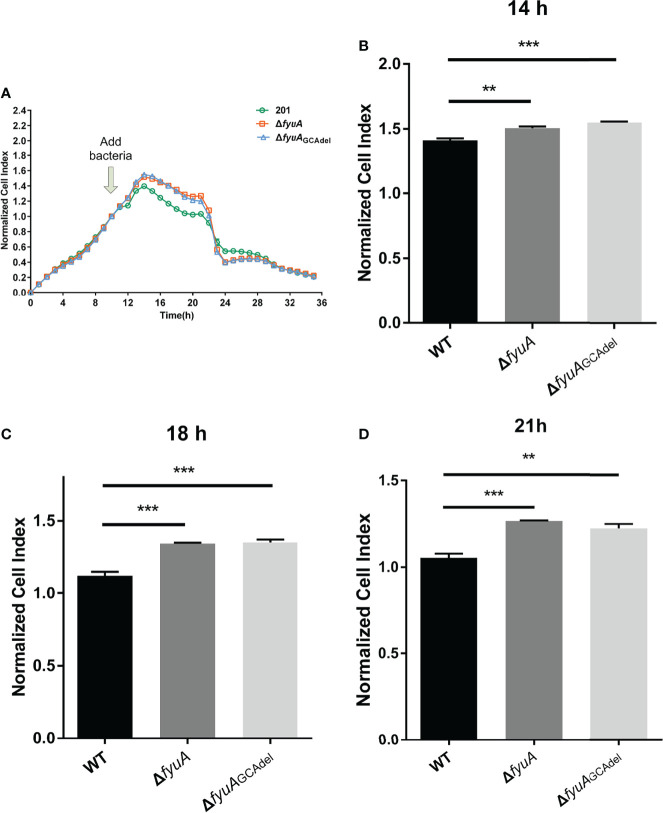
Real-time cell analysis assay curves of HeLa cells infected with WT and the two mutants. **(A)** Normalized CI value curves. The bacteria were added to the cell culture at 10h. **(B)** Normalized CI values of 14h (4h post infection). **(C)** Normalized CI values of 18h (8h post infection). **(D)** Normalized CI values of 21h (11h post infection). **, p < 0.01. ***, p < 0.001.

RTCA can monitor infected HeLa cells inoculated onto a gold matrix by measuring the cell index (CI), an indicator that reflects cellular changes, including cell adhesion and cell number (Roshan Moniri et al., 2015; Cetin and Topcul, 2019). The CI readouts of the WT strain infected HeLa cells were significantly lower than those of the mutant strains infected cells infected with the mutant strains from 2h to 9h post infection (12h to 21h in the RTCA assay) (**Figure 2**). This result shows that the cytotoxicity of Δ*fyuA* and Δ*fyuA*_GCAdel_ toward HeLa cells was attenuated, thus indicating that the *fyuA* gene is involved in the cytotoxicity of *Y. pestis* toward HeLa cells.

### Prediction of Ligand Binding Sites of FyuA and FyuA_Glndel_

The finding that the deletion of only three bases in *fyuA* at positions 915–917 can lead to attenuation of *Y. pestis* virulence is interesting. In this study, we predicted the structure and ligand binding sites of FyuA and FyuA_Glndel_ (glutamine at position 305 of the FyuA protein was absent) using I-TASSER and focused on differences in ligand binding sites between them. Functional templates of the target protein were comprehensively scored using COFACTOR and COACH based on the I-TASSER structure prediction. The predicted functional templates with the top five C-scores (C-score range of 0–1, where a higher score indicates a more reliable prediction) of the ligand binding sites of FyuA and FyuA_Glndel_ are depicted in **Table 1**. In the top five, three unique functional templates named 2grxA, 2W76B, and 2GSKA (PDB ID) were predicted for FyuA, which were missing in FyuA_Glndel_. The fifth functional template 2GSKA binds the ligand 2GSKA00. We searched PDB and found that the functional template is the BtuB : TonB complex. TonB, which possesses a single putative transmembrane helix, a proline-rich linker region, and a periplasmic C-terminal domain, couples the inner membrane proton-motive force (pmf) to the outer membrane transporter (Wiener, 2005). TonB-dependent outer membrane transporters possess a conserved motif named Ton-box (Lundrigan and Kadner, 1986; Schramm et al., 1987), which interacts with TonB during the active transport cycle, and deletion (or certain mutations) of the Ton-box stops the transport (Bell et al., 1990). Thus, we speculated that the deletion of glutamine (Gln) on FyuA damaged the TonB ligand binding sites that are necessary for iron transport, thus leading to attenuated virulence of Δ*fyuA*_GCAdel_ mutant strains.

**Table 1:** The ligand binding site of FyuA and FyuA_Glndel_.

FyuA				FyuA_Glndel_			
C-score	PDB Hit	Lig Name*	Ligand Binding Site Residues	C-score	PDB Hit	Lig Name	Ligand Binding Site Residues
0.05	1ujwA	LIM	147, 148, 599, 611, 642, 644	0.06	1ujwA	LIM	147, 148, 598, 610, 641, 643
0.05	2grxA	FTT	214, 263, 278, 280, 301, 330	0.05	3m8bA	C8E	321, 361, 363, 373, 374, 375, 376
0.05	1nqhA	C8E	360, 378, 379, 380, 407, 409, 410	0.04	1NQHA	1NQHA00	60, 61, 62, 63, 64, 65, 67, 76, 77, 78, 79, 80, 229, 230, 231, 232, 287, 297, 544, 545, 623, 624, 625, 632
0.03	2W76B	2W76B01	562, 563, 564, 565, 567, 599, 609, 610, 611, 644, 645, 646	0.03	1nqgA	C8E	359, 377, 378, 406, 408, 409, 410
0.03	2GSKA	2GSKA00	60, 61, 62, 63, 64, 65, 67, 76, 77, 78, 79, 80, 229, 230, 231, 232, 287, 289, 296, 298, 545, 546, 624, 625, 626, 633	0.03	2FCPA	2FCPA02	218, 262, 263, 280, 302, 304, 329

*Lig Name is name of possible binding ligand.

### Mutants Showed Different Virulence Toward Mice in Different Plague Models and Iron Accelerated Lethality During Their Subcutaneous Infection

**Figure 3:**
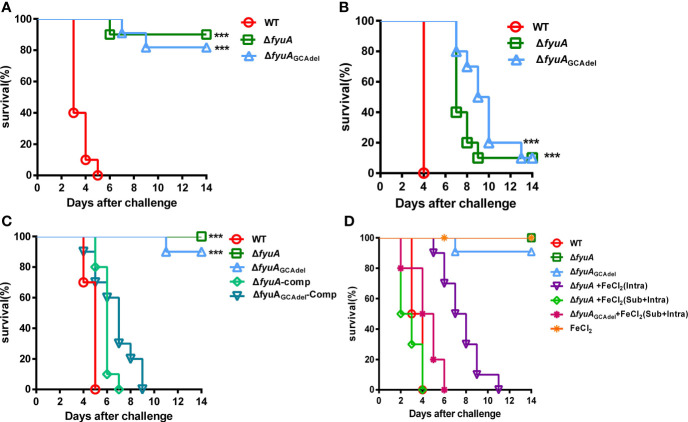
*Y. pestis* Δ*fyuA* and Δ*fyuA*_GCAdel_ mutants showed virulence attenuation in mice, and iron accelerated the mouse lethality during subcutaneous infection with Δ*fyuA* and Δ*fyuA*_GCAdel_ mutants. Each mouse of each group (n = 10) was challenged with 10^4^ CFU of bacteria. **(A)** Intraperitoneal challenges with WT, Δ*fyuA*, or Δ*fyuA*_GCAdel_. **(B)** Intravenous challenges with WT, Δ*fyuA*, or Δ*fyuA*_GCAdel_. **(C)** Subcutaneous challenges with WT, Δ*fyuA*, Δ*fyuA*_GCAdel_, and the complemented strains of the two mutants. **(D)** The WT, Δ*fyuA*, and Δ*fyuA*_GCAdel_ groups were subcutaneously challenged with WT, Δ*fyuA*, or Δ*fyuA*_GCAdel_ respectively. The FeCl_2_ groups were challenged with the mutants and were supplemented with 50 μg of FeCl_2_ intraperitoneally or subcutaneously plus intraperitoneally. ***, p < 0.001.

*Y. pestis* biovar Microtus 201 strains are highly virulent toward mice but avirulent to guinea pigs and other large animals. The *fyuA* gene plays an important role in the virulence of *Yersinia* spp. (Rakin et al., 1994; Brem et al., 2001). Based on the finding of lower cytotoxicity of Δ*fyuA*_GCAdel_ toward HeLa cells, we challenged the mice *via* three routes. In groups of BALB/c mice (n = 10) infected with WT, Δ*fyuA*, or Δ*fyuA*_GCAdel_ *via *intraperitoneal (intra) injection, Δ*fyuA* and Δ*fyuA*_GCAdel_ displayed significantly attenuated virulence [P < 0.0001, Log-rank (Mantel–Cox) Test]. Additionally, the 14-day survival rates of mice challenged with Δ*fyuA* or Δ*fyuA*_GCAdel_ mutants were 90% and 80%, respectively (**Figure 3A**). In groups of BALB/c mice (n = 10) infected with the WT or mutant strains *via* the intravenous route (septicemic plague model), the 14-day survival rate of mice injected with each mutant was 10% (**Figure 3B**). This finding differs from that of another study in which an *fyuA* mutant was fully virulent in mice when administered intravenously (Bobrov et al., 2014; Perry et al., 2015). In a previous research, *Y. pestis* strains with mutations in *fyuA* showed losses of virulence of >1.13 × 10^6^-fold and 33-fold upon subcutaneous and intranasal infections, respectively (Fetherston et al., 2010). In our research, a similar result was obtained when the mice were challenged subcutaneously (**Figure 3C**). Moreover, the complemented strains of the mutants showed virulence reversion on mice challenged through subcutaneous injection (**Figure 3C**).

The attenuation of nonpigmented KIM D27 strains, when administered subcutaneously, has long been attributed to deficient iron acquisition and metabolism, partially because intraperitoneal injection of inorganic iron appeared to restore virulence (Burrows and Jackson, 1956; Brubaker et al., 1965). This situation is also applicable to pneumonic plague models (Lee-Lewis and Anderson, 2010). The structure prediction using I-TASSER indicated that the attenuation of Δ*fyuA*_GCAdel_ may be due to the blockage of iron transportation. To confirm this result, FeCl_2_ was injected into mice throughout the experiment. The iron-treated mice were challenged with Δ*fyuA* and Δ*fyuA*_GCAdel_ subcutaneously (bubonic plague model). The mice injected with 50 μg of FeCl_2_ subcutaneously and intraperitoneally twice daily exhibited the same clinical signs as those infected with the WT strains. All mice rapidly and consistently developed lethal infection and died within 6 days (**Figure 3D**). Meanwhile, the mice injected with 50 μg of FeCl_2_ only intraperitoneally died within 14 days (**Figure 3D**), which is later than the mice infected with the WT strains. These results assert that *fyuA* is involved in the virulence of strain 201 and that the inhibition of iron uptake in the mutants might attenuate their virulence.

**Figure 4:**
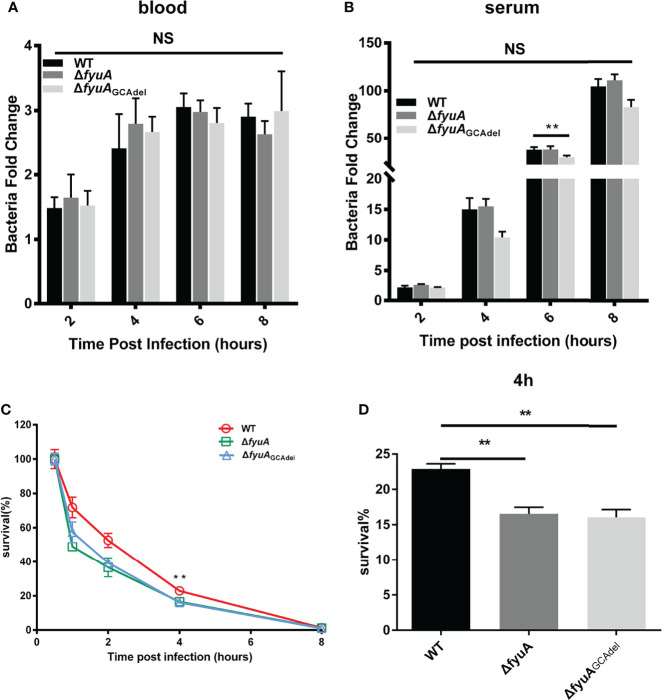
Comparison of the viabilities of WT and the mutants in blood **(A)**, serum **(B)** and RAW264.7 cells **(C, D)**. The survival rates of Δ*fyuA* and Δ*fyuA*_GCAdel_ mutant strains were lower than that of strain 201 in RAW264.7 cells but showed no significant differences in blood and serum. The experiments were performed three times independently. **, p < 0.01. NS, not significant.

The formation constant of Ybt with ferric iron is 4 × 10^36^; hence, Ybt can remove iron from transferrin and lactoferrin. Blood is abundant in iron, which is a component of hemoglobin. We speculated that the Ybt system plays a more important role than other iron uptake systems when the mice are challenged subcutaneously, in which *Y. pestis* went through an iron-deficient environment. However, other systems may be more effective in acquiring iron once bacteria have disseminated *via* the bloodstream. To prove this hypothesis, we compared the abilities of *Y. pestis* 201 and mutant strains to survive in mouse blood or serum, and their survival rates were calculated by counting the colonies after incubation in blood or serum for 0, 2, 4, 6, and 8 h. The results showed that the total bacterial counts of 201 and mutant strains in blood (**Figure 4A**) or serum (**Figure 4B**) did not differ significantly, which denoted that the deletion or mutation of *fyuA* does not affect the reproduction of *Y. pestis* in blood or serum. This finding supports our hypothesis that the Ybt system may not work in the septicemic plague model. This result is consistent with that obtained for mice challenged by intravenous injection. Because the growth and reproduction of the *fyuA* mutant strains are not affected in the blood, the virulence of the mutant strain toward mice when administered intravenously is only slightly weakened.

### Survival Rate of Δ*fyuA* and Δ*fyuA*_GCAdel_ Mutants Was Lower Than That of Strain 201 in RAW264.7 Cells

The ability of *Y. pestis* to survive in macrophages plays an important role in the early stages of host infection (Straley et al., 1984). In this study, we compared the ability of 201 and mutant strains to survive in RAW264.7 cells to explore whether the attenuation of the mutant strains is related to their weakened ability to survive in the macrophages. RAW264.7 cells were infected continuously for 8 h with Δ*fyuA*, Δ*fyuA*_GCAdel_, and WT strains at an MOI of 5. Subsequently, their survival rates were measured using a gentamycin protection assay, followed by plating the cell lysates on Hottinger’s plates. The number of live bacteria in RAW264.7 cells at 0.5 hpi was designated as the initial value, and the survival rates at 1, 2, 4 and 8 hpi were calculated by dividing the corresponding live bacterial cell number with that at 0.5 hpi. The percentage survival of 201, Δ*fyuA*, and Δ*fyuA*_GCAdel_ decreased to approximately 70%, 45%, and 55%, respectively, in RAW264.7 cells at 1 hpi. This percentage continued to decrease to approximately 50% and 35%–40% at 2 hpi. This trend of WT exhibiting higher survival rates than the mutant strains in RAW264.7 cells continued until 6 hpi; neither WT nor mutant strains were detected in the cells at 8 hpi (**Figures 4C, D**). These results indicates that *fyuA* plays an important role in the early stage of *Y. pestis* infection, and the weakened ability of the mutant strain to survive in macrophages may contribute to its attenuated virulence.

### Comparative Transcriptomics of 201 and Δ*fyuA*_GCAdel_ Cultured at 26°C or 37°C

**Figure 5:**
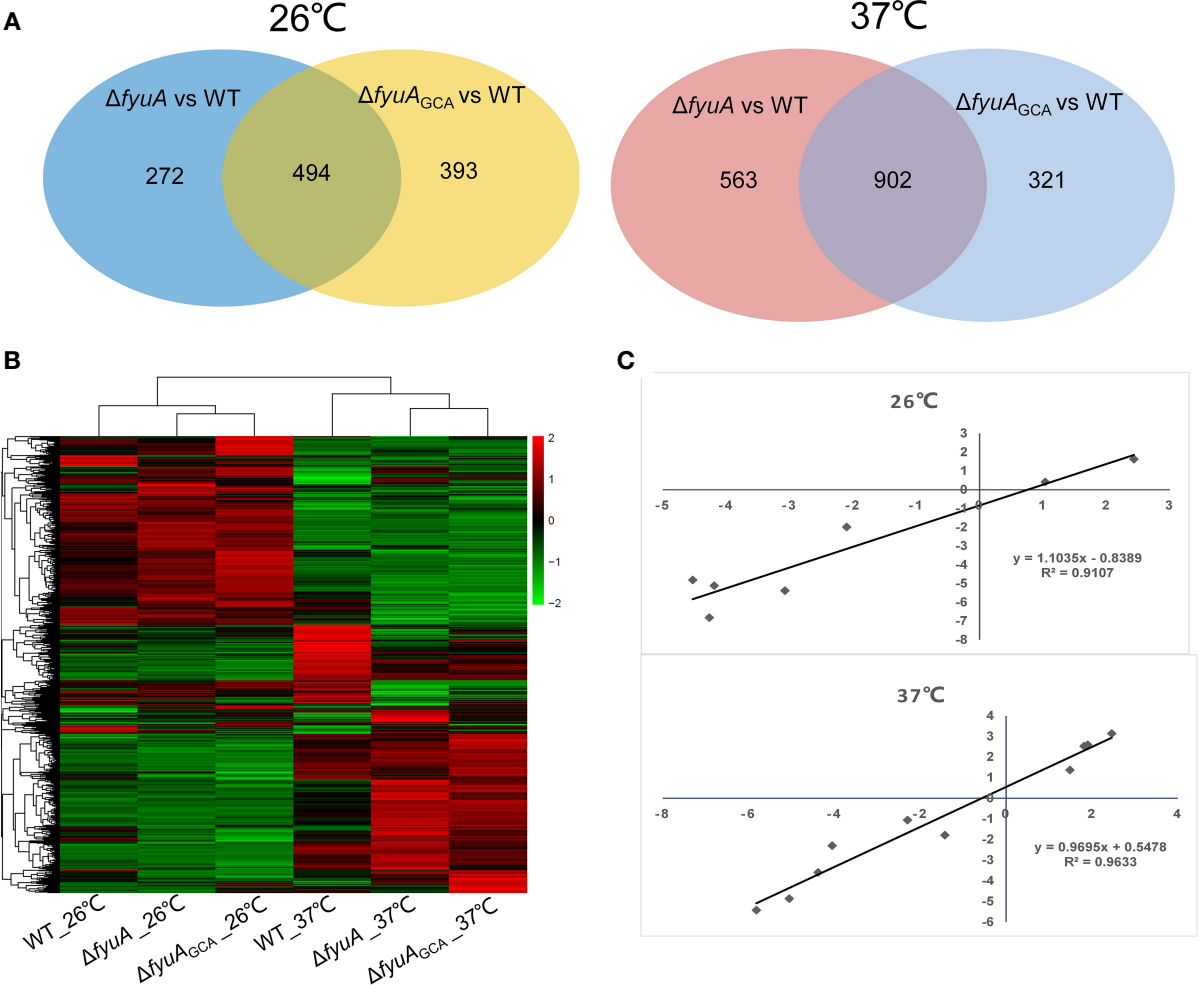
Comparative transcriptomics of strain 201 and Δ*fyuA*_GCAdel_ cultured at 26°C or 37°C. **(A)** Venn diagrams showing overlapping of the gene expression profiles between different comparison groups. **(B)** Clustering analysis of differential gene expression between WT and mutant strains. Different colors show different gene expression levels, with high expression being colored red and low expression being colored green. **(C)** Correlations among the expression levels of 17 genes measured with RNA-seq and qRT-PCR were analyzed using linear regression.

To better explore the influence of GCA deletion or *fyuA* gene knockout with GCA deletion on *Y. pestis*, we compared the transcriptomes of strain 201, Δ*fyuA*, and Δ*fyuA*_GCAdel_ cultured at 26°C or 37°C (**Figure 5**). The RNA-seq data were confirmed using the qRT-PCR analysis of several genes (**Supplementary Table 4**), and the samples used in RNA-seq library construction were used as templates in qRT-PCR. The correlation coefficients (R^2^) between the RNA-seq and qRT-PCR results were >0.90 for the RNA samples obtained at 26°C and 37°C (**Figure 5C** and **Supplementary Table 4**). These results signify the high reliability of the RNA-seq data described in this paper.

As shown in **Figure 5A**, 766 genes of ΔfyuA and 887 genes of ΔfyuAGCAdel cultured at 26°C were differentially expressed when compared with strain 201, and 494 of these genes were shared in these two mutant strains. While when cultured at 37°C, the gene numbers were 1438, 1223 and 902 respectively. The clustering heatmap shown in **Figure 5B** also revealed similar expression patterns of ΔfyuA and ΔfyuAGCAdel cultured at these two temperatures.

The results of RNA-seq showed that genes in Δ*fyuA*_GCAdel_ related to the Ybt system, such as *ybtX*, *ybtS*, *YbtQ*, *YbtU*, *YbtT*, *YbtE*, *irp2*, *irp1*, and *fyuA*, were downregulated compared with those of strain 201 at both 26°C and 37°C (**Table 2**). Thus, *fyuA* may have some potential regulatory effects on the Ybt system. Moreover, the genes of the type III secretion system (T3SS) in Δ*fyuA*_GCAdel_, such as *lcrV*, *yscA*, *yscX*, *yopT*, *yopE*, and *yopB*, were upregulated compared with those in strain 201 at 37°C (**Table 3**).

**Table 2:** The genes of Ybt system were down-regulated in Δ*fyuA*_GCAdel_ in comparison with 201-WT.

gene_id	log_2_FoldChange 26°C	log_2_FoldChange 37°C	gene_name	gene_description
YP_RS08660	-2.9	-2.5	*ybtS*	yersiniabactin biosynthesis salicylate synthase YbtS
YP_RS08655	-3.1	-2.4	*ybtX*	yersiniabactin-associated zinc MFS transporter YbtX
YP_RS08650	-2.1	-2.3	*ybtQ*	yersiniabactin ABC transporter ATP-binding/permease protein YbtQ
YP_RS08645	-2	-3.9	*ybtP*	yersiniabactin ABC transporter ATP-binding/permease protein YbtP
YP_RS08635	-4.2	-4	*irp2*	yersiniabactin non-ribosomal peptide synthetase HMWP2
YP_RS08630	-3	-2.8	*irp1*	yersiniabactin polyketide synthase HMWP1
YP_RS08625	-3.5	-2.9	*ybtU*	yersiniabactin biosynthesis oxidoreductase YbtU
YP_RS08620	-4.5	-3.1	*ybtT*	yersiniabactin biosynthesis thioesterase YbtT
YP_RS08615	-2.9	-2.6	*ybtE*	yersiniabactin biosynthesis salycil-AMP ligase YbtE
YP_RS08610	-4.3	-5	*fyuA*	siderophore yersiniabactin receptor FyuA

**Table 3:** The genes of type III secretion system were up-regulated in Δ*fyuA*_GCAdel_ in comparison with 201-WT at 37°C.

gene_id	log_2_FoldChange	gene_name	gene_description
YP_RS21370	2.1	*yerA*	YopE transcriptional regulator year
YP_RS21365	1.5	*yopE*	type III secretion system effector GTPase activator YopE
YP_RS21310	1.8	*yopT*	T3SS effector cysteine protease YopT
YP_RS21305	1.4	*sycT*	type III secretion system chaperone SycT
YP_RS21285	2.1	*-*	type III secretion system leucine rich repeat protein
YP_RS21270	1.4	*yopD*	type III secretion system translocon subunit YopD
YP_RS21265	1.5	*yopB*	type III secretion system translocon subunit YopB
YP_RS21260	1.7	*lcrH*	type III secretion system chaperone LcrH
YP_RS21255	1.8	*lcrV*	type III secretion system protein LcrV
YP_RS21250	1.8	*lcrG*	type III secretion protein LcrG
YP_RS21245	1	*lcrR*	type III secretion system regulator LcrR
YP_RS21235	1.9	*yscY*	type III secretion system chaperone YscY
YP_RS21230	1.9	*yscX*	type III secretion system protein YscX
YP_RS21225	1.5	*sycN*	type III secretion chaperone SycN
YP_RS21220	1.2	*tyeA*	type III secretion system gatekeeper subunit TyeA
YP_RS21215	1.2	*yopN*	type III secretion system gatekeeper subunit YopN
YP_RS21190	1.1	*-*	type III secretion system export apparatus protein SctR
YP_RS21160	2.5	*yscA*	type III secretion system protein YscA
YP_RS21155	1.8	*yscB*	type III secretion system chaperone YscB
YP_RS21145	1.3	*-*	type III secretion system inner membrane ring subunit SctD
YP_RS21140	1.2	*-*	YscE family type III secretion system needle protein co-chaperone
YP_RS21135	1.3	*-*	type III secretion system needle filament subunit SctF
YP_RS21130	1.2	*-*	YscG family type III secretion protein
YP_RS21110	1.5	*-*	type III secretion system sorting platform protein YscK
YP_RS21080	1.1	*yopH*	T3SS effector protein-tyrosine-phosphatase YopH
YP_RS21065	1.4	*yopJ*	type III secretion system effector acetyltransferase YopJ
YP_RS21060	1	*yopO*	T3SS effector protein kinase YopO/YpkA

## Discussion

To investigate the role of *fyuA* in the virulence and pathogenicity of *Y. pestis* biovar Microtus strain 201, we constructed Δ*fyuA* and Δ*fyuA*_GCAdel_ mutants. We compared the phenotypes of the two mutants and strain 201 in terms of their virulence toward mice and viability in mouse blood, serum and in RAW264.7 cells. In previous studies, the *fyuA* gene has been shown to be associated with the growth and virulence of *Y. pestis* (Heesemann et al., 1993; Rakin et al., 1994; Fetherston et al., 2010). In this paper, we have confirmed that a GCA deletion of *fyuA*, which cause a Gln_306_ deletion in FyuA could also lead to similar results as Δ*fyuA*. KIM6+ strains with *fyuA* mutation showed a significant growth defect compared with the WT strain in deferrated PMH2 with 10 μM FeCl_3_. The mutants displayed virulence losses of >1.13 × 10^6^-fold and 33-fold when the mice were injected subcutaneously and intravenously, respectively (Fetherston et al., 2010). Our findings alluded that there were no significant differences in growth between strain 201 and the two mutants in LB or TMH medium containing different types and concentrations of iron. Thus, the phenotypic difference among the strains observed in this study was not influenced by their growth.

We compared the cytotoxicity of the WT and mutant strains toward HeLa cells. The results showed that the cytotoxicity of Δ*fyuA* and Δ*fyuA*_GCAdel_ mutant strains toward HeLa cells was reduced. In mouse infection experiments, we also found that the virulence of *fyuA* mutants was attenuated, with the degree of attenuation differing among the different challenge routes. The mutant strains were highly attenuated when administered *via* the subcutaneous and intraperitoneal routes.

I-TASSER was used to predict the ligand binding site of FyuA and the mutant protein. The results implied that FyuA_Glndel_ lacks the binding site named 2GSKA, which plays an important role in combining with TonB in the functional model (Shultis et al., 2006). The inner membrane protein TonB couples the inner membrane pmf to the outer membrane, and the protein consists of a single putative transmembrane helix, a proline-rich linker region, and a periplasmic C-terminal domain (Wiener, 2005). The C-terminal domain of TonB binds directly to a conserved region, named Ton-box, in the outer membrane transporter (Shultis et al., 2006). Ton-box interacts with TonB during the active transport cycle. Several studies have asserted that mutations in Ton-box prevent the transport of iron and bacteriocins (Cascales et al., 2007). The structure of FyuA in *Y. pestis* has been characterized and possesses features typical of the TonB-dependent transporter family, including a membrane-spanning 22-strand β-barrel whose pore is blocked by a plug domain (Lukacik et al., 2012). Thus, we speculated that the mutation of FyuA_Glndel_ damaged the TonB ligand binding sites relevant to iron transport, thereby leading to the attenuated virulence of Δ*fyuA*_GCAdel_ mutant strains. Our hypothesis was confirmed by the increased lethality during subcutaneous infection with Δ*fyuA*_GCAdel_ mutant along with iron supplementation.

Iron uptake from the host is vital for the pathogenicity of *Y. pestis*. The *fyuA* gene is located on the HPI island of the chromosomal *pgm* locus. In previous studies, both Ybt biosynthetic/transport mutants and Δ*pgm* strains have been shown to be essentially avirulent in a bubonic plague model (Fetherston et al., 2010). Restoration of the virulence of the Δ*pgm* mutant strains in bubonic and pneumonic plague models was achieved *via* the intraperitoneal injection of inorganic iron (Burrows and Jackson, 1956; Brubaker et al., 1965; Lee-Lewis and Anderson, 2010). In our study, the virulence of the *fyuA* mutant strains in the bubonic plague model was also restored by iron supplementation to the mice. This observation reminded us that the different degrees of attenuation of the mutant strains when administered *via* different routes may be related to the uneven distribution of iron in the mice. Furthermore, strain 201 and the mutants had similar viabilities in mouse blood, which may be the reason why the mutants exhibited only a mild attenuation intravenously. Une and Brubaker have also proven that the Δ*pgm* mutant is fully virulent in mice using a septicemic plague model (Burrows and Jackson, 1956; Une and Brubaker, 1984). Consequently, we speculated that the Ybt system acquiring iron from transferrin and lactoferrin plays a more important role than the other systems in the bubonic plague model. However, other iron uptake systems may be more effective in acquiring iron once the bacteria have disseminated *via* the bloodstream. This point may be supported by the reported finding that a researcher suffering from hemochromatosis was infected with an attenuated nonpigmented *Y. pestis* (designated UC91309) and died of septicemic plague (Frank et al., 2011).

The results of RNA-seq showed that the expression levels of the related genes in the Ybt system of Δ*fyuA*_GCAdel_ were significantly downregulated. The decreased synthesis of Ybt also inhibited iron uptake, which may be responsible for Δ*fyuA*_GCAdel_ attenuation. The mechanisms behind the involvement of FyuA in regulating the Ybt system need to be further explored. Additionally, the expression of genes encoding components of the T3SS in the mutant strains was upregulated in comparison with that of WT at 37°C, thus indicating that FyuA also has a potential role in regulating the T3SS.

Considering that the viability of *Y. pestis* in macrophages has a vital role in the early stages of host infection (Straley et al., 1984), we compared the survivability of 201 and mutant strains in RAW264.7. The results showed that the survival rates of Δ*fyuA* and Δ*fyuA*_GCAdel_ mutants were lower than that of strain 201, which contributed to the attenuation of Δ*fyuA* and Δ*fyuA*_GCAdel_ mutant strains.

## Data Availability Statement

The datasets presented in this study can be found in online repositories. The names of the repository/repositories and accession number(s) can be found below: https://www.ncbi.nlm.nih.gov/geo/, GSE198937.

## Ethics Statement

The animal study was reviewed and approved by Beijing Institute of Microbiology and Epidemiology.

## Author Contributions

YS, RY, and ZQ conceived and designed the study. YLC, KS, XC, RL, QZ, YH, and YT performed the experiments and data analysis. YLC, YJC, and YB performed bioinformatics analysis. YLC, KS, RY, and YS wrote the original draft. YLC, KS, RY, ZD, ZQ, and YS reviewed and revised the manuscript. All authors have read and agreed to the published version of the manuscript.

## Funding

This research was supported by National Natural Science Foundation of China (81660349, 31470138), and National Health Commission (2019PT310004).

## Conflict of Interest

The authors declare that the research was conducted in the absence of any commercial or financial relationships that could be construed as a potential conflict of interest.

The reviewer XW declared a shared affiliation with the author YLC to the handling editor at the time of review.

## Publisher’s Note

All claims expressed in this article are solely those of the authors and do not necessarily represent those of their affiliated organizations, or those of the publisher, the editors and the reviewers. Any product that may be evaluated in this article, or claim that may be made by its manufacturer, is not guaranteed or endorsed by the publisher.

## Supplementary Material

Supplementary Figure 1 Identification of the Δ*fyuA* and Δ*fyuA*_GCAdel_ mutants. **(A)** PCR analysis of the mutant strain Δ*fyuA*. Lanes 1 and 4: Negative control. Lanes 2 and 5: The mutant strain Δ*fyuA* showed no PCR product using internal primers *fyuA*-seqF/R and *fyuA*-seqF/R-1. Lanes 3 and 6: Strain 201 showed 431 bp and 1062 bp PCR products using primers *fyuA*-seqF/R and *fyuA*-seqF/R-1, respectively. **(B)** Sequencing analysis of *fyuA* of the Δ*fyuA*_GCAdel_ strain. PCR using the *fyuA*-seqF/R primers was performed for amplification; the amplified products were confirmed using DNA sequencing and aligned with the *fyuA* gene using SnapGene to confirm the successful construction of Δ*fyuA*_GCAdel_.
