# Supplementary figures and images for: Attenuation of Yersinia pestis fyuA Mutants Caused by Iron Uptake Inhibition and Decreased Survivability in Macrophages
Source: Front Cell Infect Microbiol. 2022 May 4;12:874773. doi: 10.3389/fcimb.2022.874773 (PMC9114763; doi:10.3389/fcimb.2022.874773)

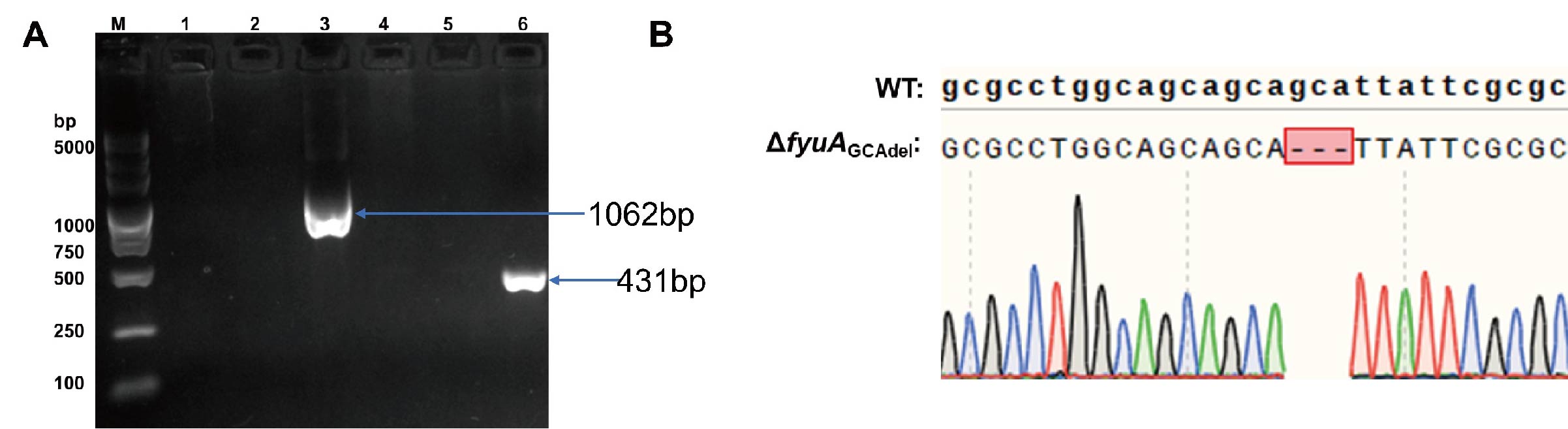

Supplement: Supplementary Figure 1 — Identification of the ΔfyuA and ΔfyuA GCAdel mutants. (A) PCR analysis of the mutant strain ΔfyuA. Lanes 1 and 4: Negative control. Lanes 2 and 5: The mutant strain ΔfyuA showed no PCR product using internal primers fyuA-seqF/R and fyuA-seqF/R-1. Lanes 3 and 6: Strain 201 showed 431 bp and 1062 bp PCR products using primers fyuA-seqF/R and fyuA-seqF/R-1, respectively. (B) Sequencing analysis of fyuA of the ΔfyuA GCAdel strain. PCR using the fyuA-seqF/R primers was performed for amplification; the amplified products were confirmed using DNA sequencing and aligned with the fyuA gene using SnapGene to confirm the successful construction of ΔfyuA GCAdel. [file Image_1.jpeg]
