# Supplementary material for: Attenuation of Yersinia pestis fyuA Mutants Caused by Iron Uptake Inhibition and Decreased Survivability in Macrophages
Source: Front Cell Infect Microbiol. 2022 May 4;12:874773. doi: 10.3389/fcimb.2022.874773 (PMC9114763; doi:10.3389/fcimb.2022.874773)
Supplement: Supplementary file 2 [file Table_1.docx]

**Supplementary Table 1.** Bacterial strains and plasmids used in this study.

| **Strain or plasmid** | **Genotype** | **Reference or source** |
| --- | --- | --- |
| ***E. coli*** |  |  |
| S17λ*pir* | Tp^r^ Sm^r^ *recA thi pro hsdR^−^M^+^* (RP4-2-Tc::Mu: Kan^r^Tn7) *λpir* | (Simon et al., 1983; Brem et al., 2001) |
| S17-pDS132-*fyuA* | pDS132-*fyuA* was introduced into S17λpir | This study |
| S17-pDS132-*fyuA*_GCAdel_ | pDS132-*fyuA*_GCAdel_ was introduced into S17-1λpir | This study |
| ***Y. pestis*** |  |  |
| strain 201 | *Y. pestis* biovar Microtus strain 201, WT | (Fan Z, 1995; Song Y, 2004) |
| Δ*fyuA* | deleted *fyuA* based on strain 201 | This study |
| Δ*fyuA*_GCAdel_ | deleted GCA three-base at positions 915~917 of *fyuA* based on strain 201 | This study |
| Δ*fyuA*-Comp | Δ*fyuA* containing plasmid pACYC184-*fyuA* | This study |
| Δ*fyuA*_GCAdel_-Comp | Δ*fyuA*_GCAdel_ containing plasmid pACYC184-*fyuA* | This study |
| **plasmids** |  |  |
| pDS132 | Suicide vector, Derived from pCVD442, without IS1 sequences. *bla* gene replaced by the *cat* gene | (Philippe N, 2004) |
| pACYC184 | Cloning vector, Cm^r^ Tet^r^ | (Chang, 1978) |
| pDS132-*fyuA* | Upstream (524bp) and downstream (471bp) homology arm of *fyuA* were inserted into pDS132 |  |
| pDS132-*fyuA*_GCAdel_ | *fyuA* gene deleted GCA bases at 915~917 was inserted into pDS132 |  |
| pACYC184-*fyuA* | *fyuA* gene was inserted into pACYC184, used for the complement of *fyuA* | This study |

Brem, D., Pelludat, C., Rakin, A., Jacobi, C.A., and Heesemann, J. (2001). Functional analysis of yersiniabactin transport genes of *Yersinia enterocolitica*. *Microbiology (Reading)* 147(Pt 5)**,** 1115-1127. doi: 10.1099/00221287-147-5-1115.

Chang, A. (1978). Construction and characterization of amplifiable multicopy DNA cloning vehicles derived from the P15A cryptic miniplasmid. *Journal of Bacteriology* 134.

Fan Z, L.Y., Wang S, Jin L, Zhou X, Liu J, Zhang Y, Li F. (1995). Microtus brandti plague in the Xilin Gol Grassland was inoffensive to humans. *Chin J Control Endem Dis* 10**,** 56-57.

Philippe N, A.J.P., Coursange E, Geiselmann J, Schneider D. (2004). Improvement of pCVD442, a suicide plasmid for gene allele exchange in bacteria. *Plasmid* 51(3)**,** 246-255. doi: 10.1016/j.plasmid.2004.02.003.

Simon, R., Priefer, U., and Pühler, A. (1983). Genetic Engineering: Transposon Mutagenesis in Gram Negative Bacteria. *Bio/Technology*.

Song Y, T.Z., Wang J, Wang L. (2004). Complete genome sequence of *Yersinia pestis* strain 91001, an isolate avirulent to humans. *DNA Res* 11(3)**,** 179-197. doi: 10.1093/dnares/11.3.179.
