# Supplementary material for: Attenuation of Yersinia pestis fyuA Mutants Caused by Iron Uptake Inhibition and Decreased Survivability in Macrophages
Source: Front Cell Infect Microbiol. 2022 May 4;12:874773. doi: 10.3389/fcimb.2022.874773 (PMC9114763; doi:10.3389/fcimb.2022.874773)
Supplement: Supplementary file 3 [file Table_2.docx]

**Supplementary Table 2.** Primers used in the gene knockout and complement.

| Primer | Primer sequence (5’-3’) | Primer function |
| --- | --- | --- |
| Pre-*fyuA*-F | agaggtacc**gcatgc**TTACCGCACAGGCGACAATG | Amplify the upstream homology arm of *fyuA* (524bp) |
| Pre-*fyuA*-R | TAAAAGGGATACCTTTGTTGTGAGTCCCTG |  |
| Post-*fyuA*-F | AAGGTATCCCTTTTACACCACTAG | Amplify the downstream homology arm of *fyuA* (471bp) |
| Post-*fyuA*-R | ctcgatatc**gcatgc**TTTTACTGAACCCTAACTTTTG |  |
| pDS132-F | GTTTCTGTTGCATGGGCATAAAG | Identify whether the recombinant vector is constructed successfully |
| pDS132-R | AACAAGCCAGGGATGTAACG |  |
| *fyuA*-seqF | GCTTTCCACCAACACCATCCAG | *fyuA* internal primer, to identify whether the knockout is successful (1062bp) |
| *fyuA*-seqR | TCAAGACCCGCAGTAGGCACGA |  |
| *fyuA*-seqF-1 | ATAGGGAATGTGAAACTGCGTCTG | *fyuA* internal primer, to identify whether the knockout is successful (431bp) |
| *fyuA*-seqR-1 | GTCGGCATGTCGTAGGCTGAAT |  |
| 184-*fyuA*-F | ATCATCGAT**AAGCTT**ctgactatcagcagttgc | Amplify the *fyuA* gene |
| 184-*fyuA*-R | CGGCGTAGA**GGATCC** aggttgtttgtctgtgct |  |
| pACYC184-F | TGAAGTCAGCCCCATACG | Identify whether the pACYC184-*fyuA* is constructed successfully |
| pACYC184-R | ATACCCACGCCGAAACAA |  |

∗The underlined bases indicate the restriction enzyme sites.
