# Supplementary material for: Attenuation of Yersinia pestis fyuA Mutants Caused by Iron Uptake Inhibition and Decreased Survivability in Macrophages
Source: Front Cell Infect Microbiol. 2022 May 4;12:874773. doi: 10.3389/fcimb.2022.874773 (PMC9114763; doi:10.3389/fcimb.2022.874773)
Supplement: Supplementary file 4 [file Table_3.docx]

**Supplementary Table 3.** Primers used for qRT-PCR.

| Primer | Primer sequence (5’-3’) | Primer function |
| --- | --- | --- |
| *ybtT*-qPCR-F | GCACGCCTGCGCTGTTATTGTG | qPCR amplification of *ybtT* |
| *ybtT*-qPCR-R | CGGGCTTGTTGAATGGGATAGAAAT | qPCR amplification of *ybtT* |
| *fyuA*-qPCR-F | GATAAATCCAGTACACAATATCACG | qPCR amplification of *fyuA* |
| *fyuA*-qPCR-R | TCAAGACCCGCAGTAGGCACGA | qPCR amplification of *fyuA* |
| *irp2*-qPCR-F | CGAGGTGGTTGGCGAGTCTGTC | qPCR amplification of *irp*2 |
| *irp2*-qPCR-R | CAGGAGAAGGGTGGGTGAAGAGGT | qPCR amplification of *irp*2 |
| *ybtX*-qPCR-F | AAATACACCTCACCCGGCAGAGC | qPCR amplification of *ybtX* |
| *ybtX*-qPCR-R | TCAGCGTGGCGTAAAGACAGACC | qPCR amplification of *ybtX* |
| *ybtQ*-qPCR-F | GCGGCAGGGCGGGTGAAATGAA | qPCR amplification of *ybtQ* |
| *ybtQ*-qPCR-R | CAGCGGCGTGACAATGGTGAGC | qPCR amplification of *ybtQ* |
| *lcrV*-qPCR-F | ACCATTCAGGTGGATGGGAGCG | qPCR amplification of *lcrV* |
| *lcrV*-qPCR-R | TCCGAGCAGGTGGTGGCAAAGT | qPCR amplification of *lcrV* |
| *yopE*-qPCR-F | CGCCTGTTTGTGGTATTCCCTT | qPCR amplification of *yopE* |
| *yopE*-qPCR-R | AATTTCTGCATCTGTTGCGCCAGCC | qPCR amplification of *yopE* |
| *yscA*-qPCR-F | AATTTCAACGAAACATAGAACA | qPCR amplification of *yscA* |
| *yscA*-qPCR-R | CATCCAGCGGCGAAACAATACTGTT | qPCR amplification of *yscA* |
| *cysT*-qPCR-F | AGAGTACGAAGAAGCCGCAGAA | qPCR amplification of *cysT* |
| *cysT*-qPCR-R | GCGATAAAGATAACCGCACCAA | qPCR amplification of *cysT* |
| *tauB*-qPCR-F | TGAGTAAAGAACAGCGGCGGGTAA | qPCR amplification of *tauB* |
| *tauB*-qPCR-R | GCAATAAACGCGGATCGACAGC | qPCR amplification of *tauB* |
| *aspA*-qPCR-F | TCTGAACAAATGCCAGTCCACC | qPCR amplification of *aspA* |
| *aspA*-qPCR-R | CGGCATCCTGTAATTGGGTACGGCC | qPCR amplification of *aspA* |
| *ssuC*-qPCR-F | CCATTGTTCTGTATGCCTTACTCG | qPCR amplification of *ssuC* |
| *ssuC*-qPCR-R | TCATTGCGCTTCTCCTGATTTT | qPCR amplification of *ssuC* |
| *glpB*-qPCR-F | AGCCAGCCATTATCGGCACTCA | qPCR amplification of *glpB* |
| *glpB*-qPCR-R | CCTCGGCTTCCTGTACCAACTC | qPCR amplification of *glpB* |
| 16S-qPCR-F | GCCACACTGGAACTGAGACACG | qPCR amplification of 16S |
| 16S-qPCR-R | CGCTGAAAGTGCTTTACAACCC | qPCR amplification of 16S |
