# Supplementary material for: Attenuation of Yersinia pestis fyuA Mutants Caused by Iron Uptake Inhibition and Decreased Survivability in Macrophages
Source: Front Cell Infect Microbiol. 2022 May 4;12:874773. doi: 10.3389/fcimb.2022.874773 (PMC9114763; doi:10.3389/fcimb.2022.874773)
Supplement: Supplementary file 5 [file Table_4.docx]

**Supplementary Table 4.** Correlation coefficients between RNA-seq analysis and qRT-PCR results were analyzed.

| **gene_id** | **gene_name** | **gene_description** | **log_2_ Ration in RNA-seq（Δ*fyuA*_GCAdel_ /201-WT）** | **log_2_ Ration in qRT-PCR（Δ*fyuA*_GCAdel_ /201-WT）** | **Temperature** |
| --- | --- | --- | --- | --- | --- |
| YP_RS00945 | *tauB* | taurine ABC transporter ATP-binding subunit | -5.817017659 | -5.428114334 | 37 ℃ |
| YP_RS08610 | *fyuA* | siderophore yersiniabactin receptor FyuA | -5.048878171 | -4.861652288 | 37 ℃ |
| YP_RS13705 | *cysT* | sulfate/thiosulfate ABC transporter permease CysT | -4.379323785 | -3.592707048 | 37 ℃ |
| YP_RS08635 | *irp2* | yersiniabactin non-ribosomal peptide synthetase HMWP2 | -4.048453577 | -2.296436258 | 37 ℃ |
| YP_RS08650 | *ybtQ* | yersiniabactin ABC transporter ATP-binding/permease protein YbtQ | -2.29455282 | -1.057751572 | 37 ℃ |
| YP_RS02595 | *aspA* | aspartate ammonia-lyase | -1.425890447 | -1.787231357 | 37 ℃ |
| YP_RS21365 | *yopE* | type III secretion system effector GTPase activator YopE | 1.487005687 | 1.369845677 | 37 ℃ |
| YP_RS21255 | *lcrV* | type III secretion system protein LcrV | 1.817125889 | 2.52357971 | 37 ℃ |
| YP_RS21160 | *yscA* | type III secretion system protein YscA | 2.459938696 | 3.1366198 | 37 ℃ |
| YP_RS21230 | *yscX* | type III secretion system protein YscX | 1.902621427 | 2.592108598 | 37 ℃ |
| YP_RS08650 | *ybtQ* | yersiniabactin ABC transporter ATP-binding/permease protein YbtQ | -2.101059748 | -1.986831791 | 26 ℃ |
| YP_RS08655 | *ybtX* | yersiniabactin-associated zinc MFS transporter YbtX | -3.074345333 | -5.377942625 | 26 ℃ |
| YP_RS08635 | *irp2* | yersiniabactin non-ribosomal peptide synthetase HMWP2 | -4.190475954 | -5.117732548 | 26 ℃ |
| YP_RS08610 | *fyuA* | siderophore yersiniabactin receptor FyuA | -4.26631023 | -6.820188723 | 26 ℃ |
| YP_RS08620 | *ybtT* | yersiniabactin biosynthesis thioesterase YbtT | -4.528130133 | -4.815972939 | 26 ℃ |
| YP_RS20410 | *ssuC* | aliphatic sulfonate ABC transporter permease SsuC | 1.03454088 | 0.41314336 | 26 ℃ |
| YP_RS16855 | *glpB* | glycerol-3-phosphate dehydrogenase subunit GlpB | 2.433441382 | 1.620455339 | 26 ℃ |
